# Cervical vertebral malformations in 9 dogs: radiological findings, treatment options and outcomes

**DOI:** 10.1186/s13620-019-0141-9

**Published:** 2019-04-23

**Authors:** Ricardo Fernandes, Noel Fitzpatrick, Clare Rusbridge, Jeremy Rose, Colin J. Driver

**Affiliations:** 1 Fitzpatrick Referrals, Halfway Lane, Eashing, Godalming, GU7 2QQ UK; 2 School of Veterinary Medicine, Faculty of Health & Medical Sciences, Vet School Main Building (VSM), Daphne Jackson Road, Guildford, Surrey GU2 7AL UK

**Keywords:** Axial rotatory displacement, Pseudoarthrosis, Klippel-Feil, Atlantoaxial, C2-C3, Fused vertebrae

## Abstract

**Background:**

Disregarding atlantoaxial instability in toy breed dogs associated with dens malformation and cervical spondylomyelopathy; cervical vertebral malformations are rare and poorly characterised in veterinary medicine and consequently treatment strategies and clinical outcome are sparsely documented.

**Results:**

Electronic clinical records at our veterinary referral hospital between April 2009 and November 2018 were searched for patients presented with cervical myelopathy secondary to an underlying suspected vertebral malformation/instability. Nine dogs met the inclusion criteria. Two dogs were diagnosed with atlantoaxial pseudoarthrosis, two dogs with a syndrome similar to Klippel-Feil in humans, two dogs with congenital cervical fusion, two dogs with congenital C2-C3 canal stenosis and deficiencies of the dorsal arch of the atlas and laminae of the axis and one with axial rotatory displacement. Tetraparesis, proprioceptive deficits, cervical hyperesthesia and cervical scoliosis were the most common clinical signs. The axis was the most commonly affected vertebrae (8/9 patients). Patients diagnosed with Klippel-Feil-like Syndrome were the younger (average of 262.5 days) and patients diagnosed with fused vertebrae the oldest (average of 2896 days) in our studied population (average of 1580.8 days).

**Conclusion:**

Cervical vertebral malformations are rare, or alternatively, being underdiagnosed in veterinary medicine. Patients diagnosed with Klippel-Feil-like Syndrome had a successful medium and long-term outcome with conservative management. Surgical treatment was often indicated for the other conditions presented in this study due to spinal instability and/or myelopathy. Stabilisations via ventral approaches were revealed to be safe. Multicentre and prospective studies are necessary in veterinary medicine to better characterise clinical outcomes in cervical vertebral malformations.

## Background

Cervical vertebral malformations (CVM) can be associated with spinal deformities leading to various syndromes and clinical presentations [1]. Many congenital abnormalities of the cervical spine are asymptomatic; the true incidence is likely to be underreported in both veterinary and human medicine [2]. It is estimated that up to 5% of human foetuses have vertebral anomalies [3]. In addition, anomalies of the cervical spine in paediatric human patients often coexist with skeletal dysplasias, connective tissue disorders and inherited metabolic disorders [3, 4]. There are very few reports on CVM if atlanto-axial subluxation in toy breed dogs and cervical spondylomyelopathy are excluded. This is the first study describing different CVM including: radiological findings; treatment options; and long term outcome. Review of the literature revealed a large number of similarities between CVM in Humans and the cases reported in this study.

## Methods

Electronic clinical records of patients presented at our veterinary referral hospital between April 2009 and November 2018 were searched for cervical myelopathies associated with vertebral cervical malformations. We retrieved information regarding age, sex, breed, body weight, clinical signs, diagnostic imaging, level and type of cervical malformation, treatment modality, surgical technique, postoperative complications, medium and long-term follow-up and outcome (Table 1). Cases were numbered according to the radiological findings and presumed aetiopathogenesis. Follow-up was defined as a re-examination performed at our centre or via a telephonic conversation with the client. Medium-term follow up was defined as follow-up up to 180 days after the initial presentation at our centre. Long-term follow-up was defined as follow-up for 181 days or longer. All cervical malformations were diagnosed via high-field magnetic resonance (MR) scanning (1.5 Tesla; Siemens Symphony Tim system, Enlargen Germany), computed tomography (CT) examinations (160-slice Aquilion Prime Toshiba, Japan) and/or radiographic studies (Cuattro DR, United States). MR protocols included at least a sagittal and transverse T2-weighted (T2W) sequence and dorsal short-tau inversion recovery (STIR) sequence. Radiographs of the cervical spine included at least a neutral right lateral and a dorsoventral view. Post-operative imaging included CT scan and/or radiographs to confirm adequate implants positioning. All images were evaluated by ECVS, ECVN and ECVSMR diplomates. Patients diagnosed with atlantoaxial instability associated with abnormalities of the dens, caudal cervical spondylomyelopathy and craniocervical junction abnormalities associated with Chiari-like malformation were not included in this study.

**Table 1:** Signalment, radiological findings, treatment options, follow-ups and clinical outcome in 9 dogs with CVM

Case	Age at time of presentation (days)	Gender	Breed	Weight (kg)	Clinical signs	Level of malformation	Treatment modality	Surgical approach	Medium term follow-up (< 180 days post-operatively)	Long-term follow-up (181 or more days post-operatively)	Time of follow-up (days)	Clinical outcome
1	1451	Female entire	Miniature Schnauzer	8.2	Cervical hyperesthesia	C1-C2	Surgical	Ventral stabilisation	Resolution of clinical signs 7 days post-operatively	Resolution of the clinical signs	3190	Ambulatory, normal posture, gait and absent hyperesthesia
2	587	Male neutered	Border Collie	28	Tetraparesis, proprioceptive deficits and cervical hyperesthesia	C1-C2	Surgical	Dorsal decompression and ventral stabilisation (two stage surgery)	Normal posture and gait with occasional cervical pain. Unremarkable follow-up CT scan.	Telephonic follow-up. Reported episodes of cervical hyperesthesia every 8 weeks. Resolution of tetraparesis	1416	Ambulatory with normal posture and gait.
3	1748	Male neutered	Cocker Spaniel	17.2	Tetraparesis, thoracic limb hypermetria and hyperreflexia	C2	Surgical	Ventral stabilisation and dorsal laminectomy of C1	Neurological examination and follow-up CT scan (pin migration that required surgical removal)	CT myelogram confirmed recompression of the spinal cord at the level of C1 (left side)	484	Euthanasia 476 days after the surgery
4	144	Male entire	Staffordshire Bull Terrier	13.3	Tetraparesis, proprioceptive deficits in all four limbs and cervical hyperesthesia	C1-C2, C3-C4 and sacrum	Conservative	N/A	Moderate tetraparesis	Ambulatory with mild tetraparesis. No evidence of hyperesthesia	888	Ambulatory with normal posture and gait.
5	381	Male entire	German Shepherd Dog	34	Urinary incontinence	C2-C5 andT3	Conservative	N/A	No evidence of urinary incontinence	N/A	30	Improvement of urinary incontinence
6	1755	Female neutered	Shih Tzu	7	Tetraparesis, proprioceptive deficits and cervical hyperesthesia	C2-C3	Surgical	Ventral stabilisation	Delayed proprioception on the right thoracic limb. Unremarkable follow-up CT scan.	Telephonic follow-up. Reported resolution of tetraparesis, ataxia and cervical pain	854	Ambulatory with normal posture and gait.
7	2369	Male neutered	Shih Tzu	9.75	Tetraparesis, proprioceptive deficits	C2-C3	Surgical	Ventral stabilisation	Relapse of tetraparesis and cervical hyperesthesia. Follow-up CT scan revealed loosening of the pins at the level of C3 and break on the of the PMMA bolus at the level of C2-C3. Review surgery with stabilisation of the C1-C4 vertebral bodies with Imexx pins and PMMA. Culture of the loose pins was negative.	Six-month follow-up of the review surgery revealed improvement of the tetraparesis and absent cervical hyperesthesia. Relapse of cervical hyperesthesia 9-months after the review surgery (C6-C7 intervertebral disc protrusion)	549	Ambulatory with normal posture and gait.
8	2010	Male entire	Siberian Husky	44.6	Non-ambulatory tetraparesis, proprioceptive deficits on the thoracic limbs and cervical hyperesthesia	C4-C5	Surgical	Ventral decompression	Lost to follow-up	Lost to follow-up	11	Ambulatory with moderate tetraparesis
9	3782	Female neutered	Chihuahua	2.5	Tetraparesis, proprioceptive deficits and cervical hyperesthesia	C2-C3	Conservative (initially) then surgical	Ventral decompression	Normal posture, gait and absent cervical hyperesthesia	Recurrence of the clinical signs (gliosis at the previous surgical site, adjacent segment disease)	660	Progressive tetraparesis

## Results

Nine dogs met the inclusion criteria. Of these 9 cases, six were male (66.7%) and three were female (33.3%). The studied population included 2 Shih Tzu, 1 Cocker Spaniel, 1 Staffordshire Bull Terrier, 1 Border Collie, 1 Miniature Schnauzer, 1 German Shepherd dog, 1 Siberian Husky and 1 Chihuahua. The median age of presentation at our centre was 1580.8 days (range: 144–3782). The mean body weight was 18.3 kg (range: 2.5–44.6). The neurological examination findings of these dogs included ambulatory tetraparesis (7/9), proprioceptive deficits (7/9), cervical hyperesthesia (5/9), cervical scoliosis (5/9), hyperreflexia in all four limbs (1/9), cervical torticollis (1/9), thoracic limb hypermetria (1/9), urinary incontinence associated with urethralis muscle incompetence (1/9) and non-ambulatory tetraparesis (1/9).

All dogs had MRI of the cervical spine. Only one dog had a follow-up MR scan following post-operative neurological deterioration (case 8). Complementary imaging included cervical CT or radiographs. Eight dogs underwent CT scanning, of which four had post-operative scanning, including CT myelogram. Cervical radiographs were performed in five dogs, of which three were repeated post-operatively. The dog presented with urinary incontinence had urinalysis including culture, which was unremarkable.

The cervical vertebrae malformations were observed between the atlas and C5. The axis was the most commonly malformed vertebrae (8/9), followed by C3 (5/9). Atlas and C4 malformations were observed in 3 dogs. Two dogs had a malformed C5.

**Fig. 1:**
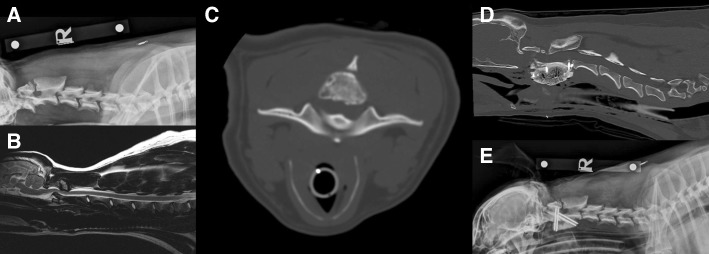
Atlantoaxial pseudoarthrosis. **a** Right lateral radiograph of the cervical spine showing pseudoarthrosis at C1-C2 and osteophyte formation between the dorsal neural arch of the atlas and the spinous process of the atlas in case 1. **b** Transverse computed tomography of the cervical spine revealing proliferative changes encroaching the vertebral canal in case 7. **c** Mid-sagittal T2W image of the cervical spine revealed cervical vertebral canal stenosis both dorsally and ventrally. The spinal cord was severely compressed at the C1-C2 level, and it was possible to identify arthrosis and beak osteophyte projecting into the vertebral canal. **d** Right lateral post-operative radiograph revealing satisfactory implant positioning. E) Mid-sagittal computed tomography of the cervical spine showing the partial odontoidectomy, ventral atlantoaxial stabilisation and significant reduction in the dorsal spinal cord compression

The imaging findings of cases 1 and 2 were characterised by atlantoaxial pseudoarthrosis with associated myelopathy. The cause of the pseudoarthrosis was unknown and may have been primary (congenital) or secondary to instability of the atlantoaxial joint. Radiological findings were characterised by spinal cord compression associated with abnormal new bone and fibrous tissue formation originating from the articular processes, which appeared abnormally incongruent and/or malformed projecting osteophytes into the spinal canal (Fig. 1a). This proliferating fibrous and bony growth was compressing the spinal cord dorsally (Fig. 1b and c). On mid-sagittal T2-weighted sequences of the cervical region of case 2, the spinal cord was additionally compressed ventrally by the odontoid process, which was dorsally angulated (Fig. 1b).

**Fig. 2:**
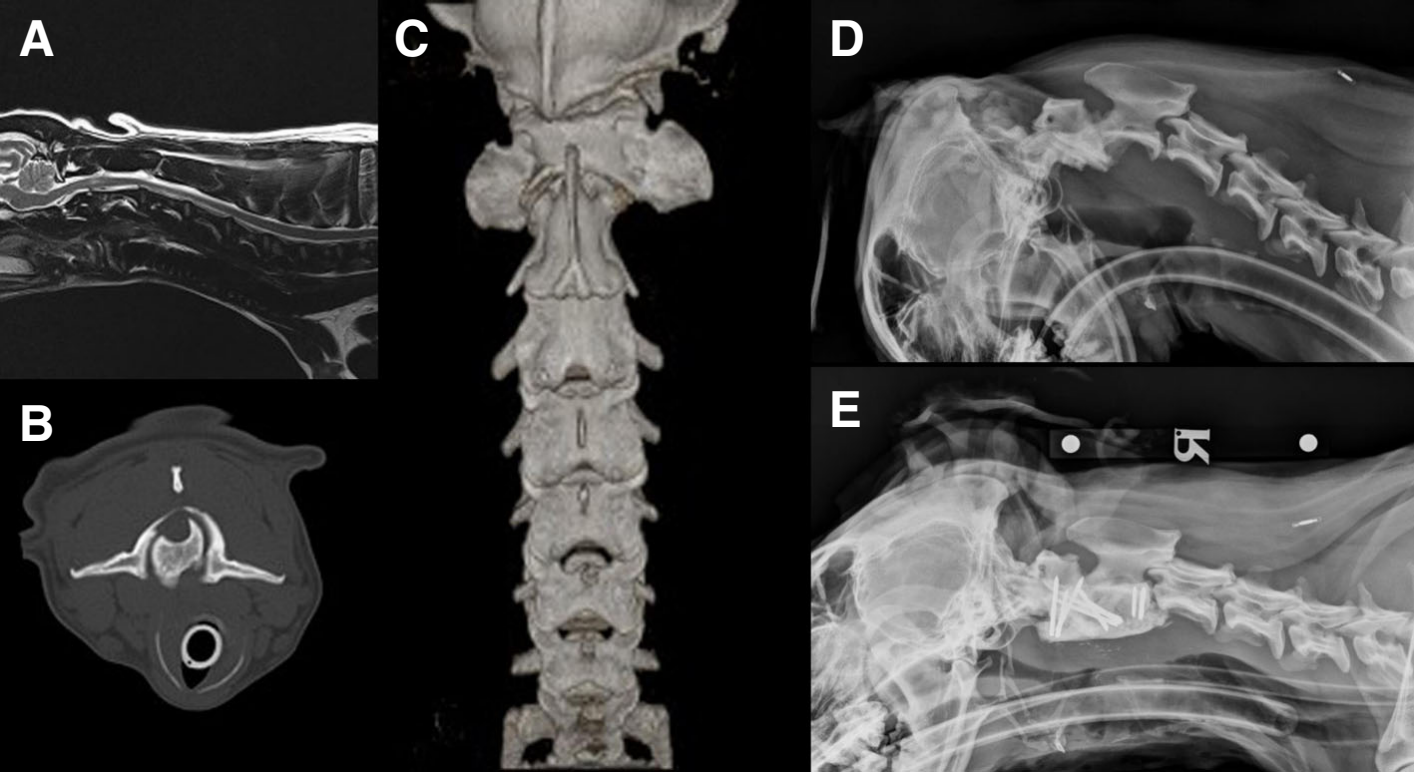
Congenital atlantoaxial rotatory displacement. **a** Mid-sagittal T2W sequence of the cervical spinal cord revealing an intramedullary spinal lesion suggestive of gliosis or vasogenic oedema. **b** Transverse computed tomography of the atlantoaxial junction revealing asymmetry of the articular facets of the axis. There is hypertrophy of the articular facet on the left side and obvious rotation of the atlantoaxial joint. **c** Dorsal view of a 3D CT-reconstruction of the cervical spine showing atlantoaxial rotation. **d** Flexed right lateral radiograph of the cervical spine demonstrating lack of obvious atlantoaxial instability. **e** Right lateral post-operative radiograph of the cervical spine revealing an adequate implant positioning

Case 3 was diagnosed with a congenital atlantoaxial rotatory displacement (CARD). Advanced imaging revealed bilateral facet subluxation and spinal cord compression secondary to marked articular facet hypertrophy, especially on the left side (Fig. 2b and c). There was an intramedullary, focal and hyperintense lesion on T2-weighted sequences at the level of C1-C2 (Fig. 2a). The odontoid process was laterally displaced to the left side indicating laxity of the transverse ligament. However, no spinal cord compression associated with the odontoid process could be observed on flexed radiographic views of the neck (Fig. 2d).

**Fig. 3:**
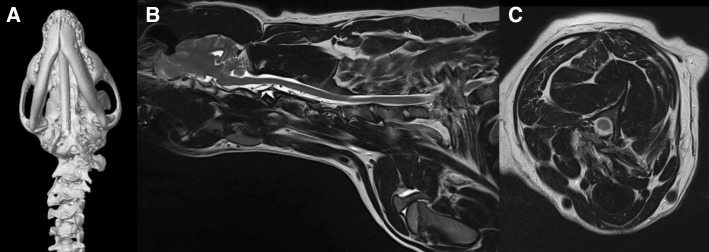
Klippel-Feil-like Syndrome. **a** Ventral view of a 3D CT-reconstruction of the cervical spine and skull showing complete absence of the left transverse process of the atlas and an asymmetric fusion of C3 and C4 in case 4. **b** Parasagittal T2W magnetic resonance sequence of the cervical spinal cord showing fusion of C2, C3 and C4. **c** Axial angular malformations and soft tissues contortion

Cases 4 and 5 were diagnosed with Klippel-Feil – like Syndrome (KFS). They were radiologically more complex and had several vertebral malformations. In case 4, three dimensional (3D) CT reconstructions revealed a failure of the ossification of the left neural arch of the atlas, with a complete absence of the left transverse process and an absent articulation between the atlas and the occipital condyle (Fig. 3a). The axis was malformed with an incomplete dens and the atlantoaxial junction appeared to be non-functional. C3 and C4 were fused asymmetrically. Sagittal MRI sequences of the cervical spinal cord at the level of C1, showed an obvious and well-demarcated intramedullary and hyperintense spinal cord lesion on T2-weighted sequences; suggesting chronic spinal cord compression with atrophy, gliosis and vasogenic oedema. This patient had eight lumbar vertebrae with failure of ossification of the sacral vertebrae dorsally and lateral deviation to the right side. Additionally, there was an absence of the right rib of T12 which was fused with the rib of T13, and the ribs of T10 and T11 were partially fused. In case 5, MRI of the spine revealed multiple, complex vertebral malformations resulting in severe cervicothoracic torticollis. There was an apparent partial fusion between the caudal aspect of the body of C2, the body of C3 (which was small without well-formed neural arches) and C4 that also had a partially dysplastic vertebral body. The C4-C5 intervertebral disc was only partially formed and the dorsal laminae were fused (Fig. 3b). Due to the variable axial angular malformations the soft tissues are contorted, with various regions of epaxial and hypaxial muscle atrophy/failure of development and/or adipose replacement (Fig. 3c). Moreover, at the level of the thoracic spine there was a failure of mid-line ossification of the T3 vertebral body resulting in median aplasia (hemivertebrae) and severe kyphoscoliosis. The imaging studies of both patients included the entire neuraxis.

**Fig. 4:**
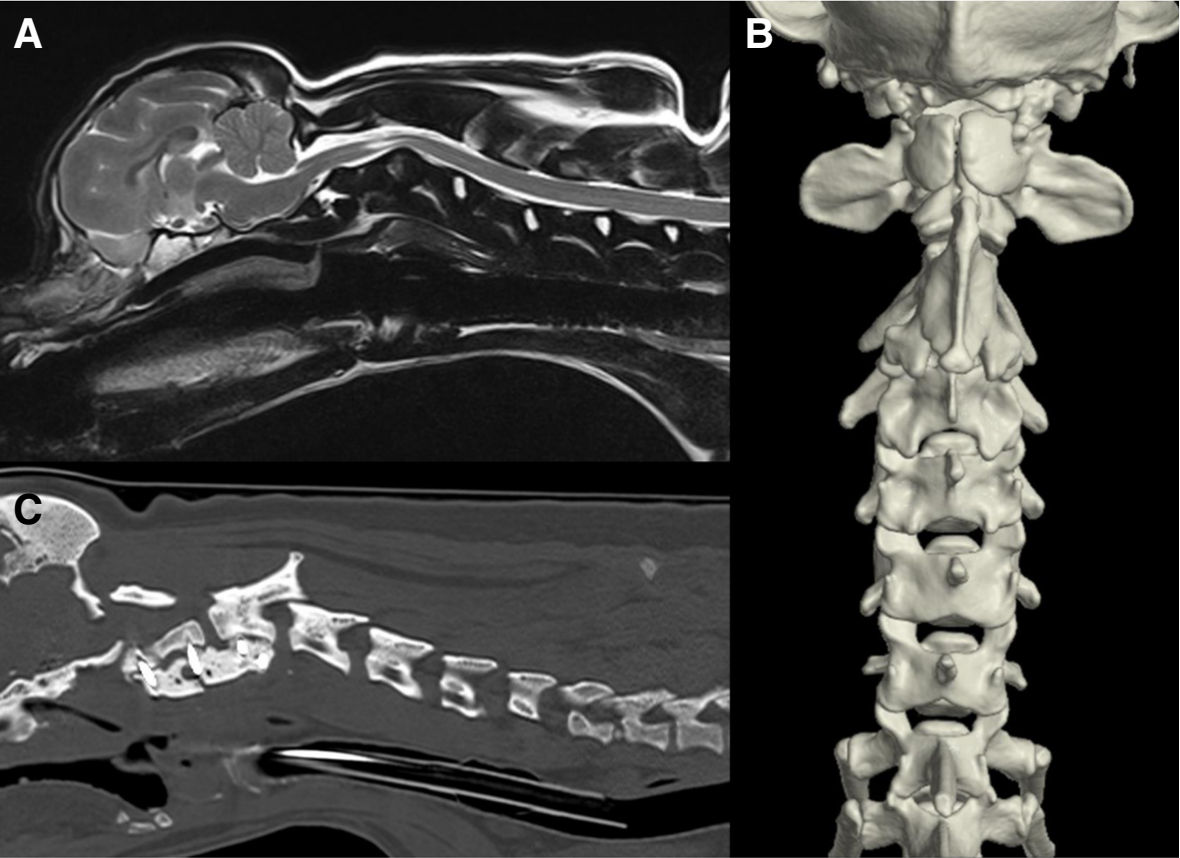
Congenital C2-C3 vertebral canal stenosis and deficiencies of the dorsal arch of the atlas and laminae of the axis. **a** Mid Sagittal T2W image of the cervical spinal cord showing ventral compression at the level of C2-C3 and an intramedullary hyperintense lesion relative to the spinal cord parenchyma. **b** Dorsal view of 3D CT-reconstruction where it was possible to observe incomplete fusion of the dorsal neural arch of the atlas, absent dorsal laminae of the axis and a short axial vertebral body (**c**) Sagittal computed tomography section of the cervical spine of case 7, where it is possible to observe failure of the PMMA bolus

Cases 6 and 7 were diagnosed with cervical myelopathy associated with congenital C2-C3 canal stenosis and deficiencies of the dorsal arch of the atlas and laminae of the axis. On MR scanning of the cervical spine, there was ventral compression of the neuraxis by the body of C3 due to dorsal displacement of C3 vertebra over C2 with an intramedullary hyperintense lesion on T2W sequences relative to the spinal cord parenchyma (Fig. 4a). 3D reconstruction of the CT sections revealed a very short axial vertebral body with absent dorsal elements, an incompletely fused dorsal neural arch of the atlas and hypertrophy of C3 spinous process (Fig. 4b).

**Fig. 5:**
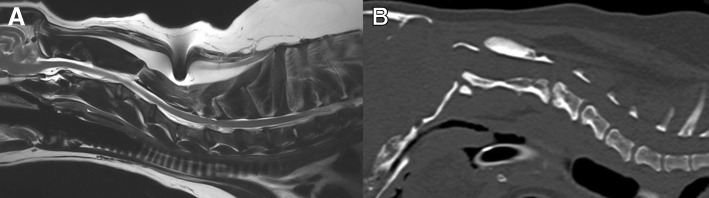
Congenital cervical fusion. **a** Intervertebral disc extrusion at the level of C5-C6, suspected to occur secondary to C4-C5 congenital cervical fusion in case 8 (adjacent segment disease). **b** Sagittal CT section of the cervical spine of case 9 showing ankylosis of the articular facets of the axis and C3 with fusion of the vertebral bodies and the dorsal lamina

Cases 8 and 9 were diagnosed with ‘blocked vertebrae’ with adjacent intervertebral disc herniation. Case 8 had ankylosis of the articular facets of C4 and C5 with fusion of the vertebral bodies and the dorsal lamina with an associated C5-C6 intervertebral disc extrusion (Fig. 5a). Case 9 had ankylosis of the articular facets of C2 and C3 with fusion of the vertebral bodies and the dorsal lamina with an associated C3-C4 intervertebral disc protrusion (Fig. 5b).

Seven dogs were treated surgically. One of these dogs underwent conservative management initially. Of the seven dogs that underwent surgical treatment, five (71.4%) had a ventral approach only and two (28.6%) had a combined dorsal and ventral approach (cases 2 and 3). Of the five dogs that had a ventral approach only, three underwent ventral stabilisation (cases 1, 6 and 7) and two had ventral decompression. Ventral decompression was performed via a ventral slot technique (cases 8 and 9). The spine was approached dorsally in two dogs and a C1-C2 partial dorsal laminectomy was performed in both. One of these dogs had a ventral stabilisation performed at the same time (Fig. 2e). The other dog had partial odontoidectomy and ventral atlantoaxial arthrodesis performed 764 days after the first procedure, due to recurrence of the clinical signs (Fig. 1d). Atlantoaxial arthrodesis was achieved by debriding the articular surfaces prior to the insertion of multiple threaded stainless steel interface pins (Imexx Interface®, United Kingdom) which were cut short and enshrouded in polymethylmethacrylate (PMMA; Stryker® Howmedica Osteonics, United States). The two dogs diagnosed with KFS were treated conservatively with gabapentin (Gabapentin®, Sandoz) and phenylpropanolamine (Propalin®, Vetoquinol).

The median time of follow-up was 898 days (range: 11–3190). One dog was lost to follow-up after discharge from our centre, 11 days after the initial presentation (case 8). Of the remaining eight, six had long term follow-up available. Immediate post-operative complications were not noticed. All dogs improved neurologically in the medium term follow-up, except two (case 7 and 9). In case 7 (cervical myelopathy associated with congenital C2-C3 canal stenosis and deficiencies of the dorsal arch of the atlas and laminae of the axis), medium-term follow-up revealed relapse of the tetraparesis. A CT scan revealed implant failure associated with breakage of the PMMA bolus (Fig. 4a). Bacterial culture of the implants was negative. This patient required a second stabilisation procedure 112 days after the initial one. The second procedure included a C1-C4 ventral stabilisation as described above. This patient was re-presented at our centre 323 days after the initial presentation with recurrent cervical hyperesthesia. A CT myelogram revealed a C6-C7 intervertebral disc extrusion that was treated surgically with a ventral slot technique. Case 9 was initially treated in a conservative manner for a C3-C4 intervertebral disc protrusion secondary to a C2-C3 cervical congenital fusion. This dog underwent a C3-C4 ventral slot 89 days after initial presentation. Resolution of the cervical hyperesthesia and improvement of the tetraparesis was noticed 21 days post-operatively. However, a relapse of the tetraparesis was reported 110 days post-operatively. A re-scan confirmed a C4-C5 intervertebral disc protrusion (adjacent segment disease). The owner declined further surgical treatment and this dog has been treated medically with prednisolone since (Prednicare®, Animalcare; approximately 0.5 mg/kg daily, orally). Case 3 showed a long-term neurological deterioration 351 days after the initial surgery and was euthanised 476 days post-operatively. In this case, a CT myelogram revealed recompression at the level of the atlantoaxial joint on the left side. Case 1 had the longest follow-up and was euthanised 3190 days after the initial presentation after being diagnosed with a humeral osteosarcoma. No recurrence of the clinical signs was noticed. The patients diagnosed with KFS were presented at younger age (mean of 262.5 days) and both patients had a higher number of malformed cervical vertebrae. Both patients were managed medically. Case 4 had a follow-up of 888 days. Neurological improvement was noticed after an initial six-week course of oral gabapentin for control of cervical hyperesthesia but moderate tetraparesis was still noticed on re-examination 228 days after the initial presentation. Case 5 was lost to follow-up 30 days after the presentation. Complete resolution of the clinical signs (urinary incontinence) with phenylpropanolamine (Propalin®, Vetoquinol) was reported. Patients diagnosed with congenital cervical fusion were the oldest (average of 2896 days) in our studied population (average of 1580.8 days).

## Discussion

In the present study we report congenital and developmental osseous abnormalities that affect the cranial and mid-cervical vertebrae in dogs resulting in neural compression and/or instability. The population included was small and heterogeneous given the rarity of these conditions and the unicentre nature of the study.

### Atlantoaxial pseudoarthrosis

In humans, pseudoarthrosis between the posterior arch of the atlas and the lamina of the axis was first reported by in 1951 [5]. Viallet [5] speculated that the contact of the atlas and axis was similar to Baastrup disease of degenerative lumbar spine, in which spinous processes contact each other closely. However, the exact mechanism of its occurrence is unclear, and reports about the relationship between such findings and clinical symptoms are scarce [6–9]. Therefore, there have been few reports showing whether the pseudoarthrosis caused any clinical symptoms such as cervical myelopathy [6–9]. Assuming that the interpretation of osteophyte formation is pseudoarthrosis, there have been only two human patients with diffuse idiopathic skeletal hyperostosis (DISH) [6, 7] and one with KFS in which osteophytes were formed at the pseudoarthrosis site of C1-C2 caused cervical myelopathy [8]. Myiakoshi and colleagues also reported atlantoaxial pseudoarthrosis associated with a chondroma in a 58-years old man [9].

The occurrence of pseudoarthrosis between the dorsal (posterior) arch of the atlas and the lamina of the axis has been recognized as one of the radiologic features occurring at the upper cervical spine [5–9] (Fig. 1a). In our canine cases, osteophytes were formed at the pseudoarthrosis between the dorsal arch of the atlas and the lamina of the axis, causing myelopathy at this level. Our cases did not reveal malformations of the dorsal arch of the atlas. Therefore, we suggest that instability may be the main trigger for the development of pseudoarthrosis, which in association with the close contact of the dorsal arch of the atlas and the lamina of the axis may well be contributory to the subsequent massive osteophyte formation. Interestingly, these abnormalities were detected in young dogs while the human case reports were described in elderly humans. In humans, pseudoarthrosis was diagnosed in combination with osteochondromas, Klippel-Feil syndrome and DISH. These underlying aetiologies were not detected in our population. Therefore, we speculate that clinically relevant atlantoaxial pseudoarthrosis in young dogs has a different (congenital or developmental) aetiology.

In the human literature, decompressive surgical interventions resulted in good outcomes [6–9]. This was another difference noticed with our canine population. In case 2, decompressive spinal cord surgery led to a relapse of the clinical signs and consequently to a ventral stabilisation. In case 1, ventral stabilization was performed in the first instance which resulted in resolution of the clinical signs (Fig. 1e). This difference suggests that instability has a more significant role in the development of the clinical signs than compression. This finding potentially suggests a different aetiology for the development of pseudoarthrosis in humans and dogs. However, definitive conclusions are impossible to reach given the small number of cases.

### (Congenital) atlantoaxial rotatory displacement (CARD)

The term atlantoaxial rotatory displacement have been used to describe a spectrum of rotational abnormalities of the atlantoaxial joint observed in the absence of major trauma [10]. The degree of rotation of the atlas depends on the integrity of the ligamentous structures [10]. Although the transverse ligament is the primary stabiliser to prevent dorsal displacement of the atlas, the alar ligaments are also vital to prevent excessive rotation of the atlas [11]. Atlantoaxial rotatory subluxation has been noted to occur in paediatric human patients with developmental disorders including juvenile rheumatoid arthritis, Down syndrome, and Morquio Syndrome [12, 13]. The underdevelopment of the cervical musculature, incomplete ossification of the odontoid process and overall ongoing immature bone ossification, a larger head to body mass ratio, and ligamentous laxity contributes to segmental hypermobility that may predispose the immature atlantoaxial region to injury [10, 12, 13]. In addition, the fulcrum of motion in the immature cervical spine is at the level of C2–C3, rather than the caudal cervical spine in adults [12, 13]. Therefore, in the paediatric patient, the atlantoaxial region is predisposed to several anatomic and biomechanical factors that increase its susceptibility to injury [12].

CARD is a radiographic diagnosis and asymmetry in size of the articular facets of C2 raises the suspicion for this diagnosis [14]. Pathologic findings identified in chronic cases include contracture of the periarticular soft tissues, interposition of fibrous tissue, osseous cross union, and adaptive changes in facet morphology [14]. Subsequently, a CT scan can help in aiding the diagnosis, especially in mild cases, and a dynamic CT study can help in distinguishing fixed from flexible deformities [14]. In humans, the normal cervical rotation is approximately 70°-80° to each side and the maximum divergence between C1 and C2 during normal rotation to either side ranges from 29° to 45° [13]. Important concerns include how C1 and C2 move relative to one another throughout the arc of rotation (C1-C2 angle or angle of separation) and whether C1 crosses over C2 [14]. This is a limitation in veterinary medicine and imaging protocols should be performed in order to establish the physiological degree of rotation in the paediatric and adult dog as recognising an early diagnosis improves outcomes.

Atlantoaxial rotatory subluxation present for longer than 6 weeks may result in chronic ligamentous changes that can increase the risk of failure to respond to conservative treatment [15]. To prevent neurologic injury, reduce the CARD deformity, prevent dislocation recurrence, and maintain sagittal balance, posterior fixation with fusion from the occiput to C4 has been described in the human literature, especially in cases of CARD associated with Klippel-Feil syndrome [13]. In our case, ventral atlantoaxial stabilisation was followed by a dorsal laminectomy for decompression of the spinal cord. It is possible that this second procedure may have contributed to some degree of instability. In the human literature, decompression has not been reported for cases of CARD. Therefore we speculate that this may have been the cause of neurological deterioration after an initial clinical improvement. However, the chronic history of tetraparesis associated with chronic ligamentous changes may have contributed to the poor long-term outcome. The authors are unsure if more extensive stabilisation may have contributed to a better long-term outcome. However, no obvious evidence for atlanto-occipital or C2-C3 dorso-ventral instability were noticed on follow-up CT myelograms. The presence of stainless steel implants prevented follow-up MRI which might have provided evidence of increased motion at the occiput-C1 articulation. Another limitation in this case was the absence of dynamic CT scans before and after clinical resolution. Therefore we could not prove that normal dynamics was restored after the initial surgery.

### Klippel-Feil syndrome

KFS is a complex, congenital condition that was initially reported in 1912 by Maurice Klippel and Andre Feil [16]. Studies have shown that 34 to 74% of KFS Human patients present with a low posterior hairline, short neck, and limited neck range of motion [16, 17]. However, the “hallmark” of this condition is the improper segmentation of cervical vertebral bodies with congenitally fused vertebrae [18]. KFS has been associated with other skeletal malformations such as rib anomalies and hemivertebrae; and scoliosis [18]. The incidence of KFS in the human population is believed to occur once in every 40,000 births [18] and up to 50% of paediatric KFS patients are symptomatic [16].

Mutations in the PAX gene family have been implicated in development of this syndrome [17]. Genetic testing has been performed in one case report of Klippel-Feil syndrome associated with Sprengel disease in a dog [19]. However, the underlying mutation responsible for the findings in that case could not be identified. In both cases presented in our study, genetic testing was not performed.

Samartzis and colleagues [16] proposed a radiographic classification scheme of congenitally fused cervical segments in patients with KFS. This classification scheme included three different criteria. Type I was defined as having a single congenitally fused cervical segment, Type II demonstrated multiple non-contiguous congenitally fused cervical segments and type III had multiple contiguous congenitally fused cervical segments. Human studies suggested a gender predilection with 60 to 70% of KFS cases occurring in females [16, 17]. However, in Gruber and colleagues retrospective study [18] this predilection could not be confirmed. Interestingly, it appeared to be a greater male predominance with increasing classification type, with the more innocuous Type I cervical fusion pattern occurring more in females, whereas Type III occurs mainly in males. The occurrence of Type II is similar in both males and females. The majority of patients were considered Type II (50%), with Type I (25%) and Type III (25%) demonstrating a similar prevalence. We present two male dogs that were classified as type III according to Samartzis scheme.

Xue and colleagues [17] concluded that in people with KFS and congenital scoliosis there was a mean of 3.29 fused segments from C2–T1. The most commonly fused segments were C2–C3, C3–C4 and C6–C7; each of which was noted in 60.7% of the patients [17]. Thirty two percent of the patients had 2 fused segments, 32.1% had 3 fused segments, 17.9% had 4 fused segments, 10.7% had 5 fused segments, and 7.1% had 6 fused segments. In our study, we observed vertebral fusion at the level of C3-C4 in case 4; and at the level of C2-C3, C3-C4 and C4-C5 in case 5, with failure of formation of the T3 vertebral body (hemivertebrae). With regard to the presence of vertebral malformations in human KFS [20], failure of segmentation was found in 3.6%, failure of formation in 17.9% and mixed deformities in 78.6%. Vertebral anomalies were also frequently seen in the thoracic region (71.4%), followed by lumbar region (17.9%) and thoracolumbar region (10.7%) [20]. Approximately 46 % of the patients with KFS exhibit rib anomalies (fused, bifid and missing ribs) [20]. Congenital scoliosis and rib anomalies were observed in both of our patients suggesting that this may be a common finding in the canine population with KFS. Also, failure of ossification of the sacral vertebrae were observed in case 3. Extra-skeletal anomalies associated with KFS included urological anomalies (35–64%), deafness (30%), congenital heart disease (14%), and Sprengel deformity (30–42%) [21]. In case 5, the urinary incontinence reported could not be clearly attributed to a myelopathy, however no urological disorder was detected and this dog was lost to follow-up after clinical improvement with phenylpropanolamine.

Aplasia of the atlas has been associated with KFS [22]. When segmentation failures of the second and third cervical vertebra occur in association with atlas abnormalities, atlantoaxial instability results due to the abnormal load placed on this motion segment [22]. Therefore, early identification of KFS is paramount to minimize the risk of neurologic injury during daily activities or operative management of such patients [22]. Interestingly, in a cohort of 32 patients with concomitant scoliosis and KFS, only 2 required surgical intervention [23]. The role of prophylactic surgical stabilization in people with isolated neck pain or without symptoms remains controversial [24]. However, persistent radicular symptoms or myelopathy may require surgical decompression and stabilization. Tetraparesis and cervical hyperesthesia were observed in case 4, where there was complete aplasia of the transverse process of the atlas on the left side. However, a gradual neurological improvement was observed. We theorised that the clinical improvement was due to the progressive cervical muscular hypertrophy characteristic of the breed, reduced head: neck ratio and presumed decreased laxity of the cranial cervical ligamentous structures. In our cases, no surgical intervention was required.

### Congenital C2-C3 canal stenosis and deficiencies of the dorsal arch

Isolated anomalies of the dorsal (posterior) elements of the axis are rare, with reported prevalence of 0.15% in the human population [25]. Posterior element anomalies may be caused by impairment of fusion between the primary ossification centres lying at the base of the articular process [25]. In humans, headache, neck pain and paresis have been reported symptoms [26]. In most cases, anomalies are found incidentally in asymptomatic patients, when the patient undergoes a radiologic assessment for neck pain, radiculopathy, neck mass or following trauma [26]. Common anomalies of the axis include total or partial absence of the odontoid process, non-fusion of the odontoid process, segmentation failure of the second and third cervical vertebrae [27]. The two cases reported here, were presented with a chronic progressive history of tetraparesis associated with cervical pain. Imaging studies revealed absence of the dorsal laminae of the axis and a cleft at the level of the dorsal neural arch of atlas. These malformations have been suggested to be a result of dysregulated genes expression, incorrect tissue interactions, cellular migration and proliferation [25]. Interestingly, both dogs were Shih Tzus and a genetic breed predisposition could be speculated. However, the small numbers do not allow us to reach a definitive conclusion.

The range of clinical and radiological manifestation in humans makes the proposition of a standard treatment protocol challenging [27, 28]. As a generally accepted rule a conservative approach in asymptomatic patients consisting of rest, administration of analgesics or muscle relaxants, cervical collars and physical therapy is advisable. However, surgery is the ideal treatment for patients with atlantoaxial subluxation [27, 28]. We postulate that the deficiencies in the dorsal laminae of C1 and C2 contributed to moderate instability and increased mechanical loading to the cervical spine, particularly at the level of C2-C3. The presence of mild intervertebral disc degeneration and mild protrusion of the C2-C3 level was indicative of this. The relatively mild protrusion may have exacerbated an already compromised spinal cord from instability, causing a more acute recent deterioration. Give the current evidence in the human literature [25, 26, 28] and the two cases reported, surgery should be the treatment of choice in patients with radiological evidence of instability, intractable long-standing neck pain and neurologic deficits. The surgical management of these cases through ventral stabilisation resulted in a successful long-term clinical outcome for both patients, despite the initial complications associated with implant failure in case 7.

### Congenital cervical fusion

A fused vertebra (‘block vertebrae’) is the result of failure of segmentation, attributed to abnormalities of the intersegmental arteries in the developing embryo [29, 30]. The malformation may occur at any point along the vertebral column and may involve part or all of a vertebrae [31, 32]. Often the fused vertebra is shorter in length than the equivalent number of normal segments, and abnormal angulation or stenosis of the vertebral canal may be present [29, 31]. A fused or ‘block’ vertebra is usually an incidental radiographic finding [31]. However, Nouri and colleagues [32] reported a relatively high prevalence (3.9%) of congenital cervical fusion (CCF) in a cohort study of human patients surgically treated with degenerative cervical myelopathy (DCM), suggesting that individuals with congenitally fused cervical vertebrae are at an increased risk of degenerative changes leading to myelopathy. The majority of these cases (78%) consisted of C2-C3 fusion of the posterior elements with complete preservation of the C2–3 intervertebral disc leading to myelopathy C3-C4 [32]. A similar anatomical localisation was observed in case 9. This was most likely related to the fact that C3-C4 was adjacent to a fused level and adjacent levels are known to experience increased biomechanical stress and accelerated degeneration [33]. The trend toward increased degeneration at levels adjacent to fused segments is consistent with observations in post-operative patients that have undergone cervical fusions [32]. Similar findings have been observed in veterinary studies [34, 35].

Interestingly, Nouri and colleagues [32] also reported that CCF patients present with symptoms of myelopathy at an older age than KFS patients (average age of 67.0 years compared with 58.8 years in the KFS group), which may be explained by the fact that a large proportion had fusions at C2–3. Anatomically, the canal at C3–4 in humans is relatively larger compared with C5-C6, making the development of myelopathy likely to require a greater degree of degeneration and thus clinical signs may take longer to manifest [32].

CCF human patients tend to be older, with a greater number of operated levels, and more frequent ossification of the posterior longitudinal ligament [32]. It makes intuitive sense that congenital fusion at C3–4 is more likely to show adjacent segment cord compression at C4–5 than C2–3 because the spinal canal is narrower at this level [33]. Furthermore, this interesting fact was also noticed in our study. Case 9, was presented with a CCF at the level of C2-C3 and DCM at the level of C3-C4. This patient was represented with DCM at the level of C4-C5 after decompressive surgery on the cranial segment. This patient was considerably older (3782 days versus 2010 days) relative to the patient number 8 that was presented with CCF at the level of C4-C5 and DCM at the level of C5-C6, suggesting that the same principle can be applied in humans and dogs. Both dogs diagnosed with fused vertebrae were considerably older than the dogs diagnosed with KFS in our study, favouring the findings observed in this human study (average of 2896 days versus 262.5 days). Despite the rarity of congenital cervical fusion, evidence suggests that CCF predisposes patients to DCM associated with adjacent segment disease.

### Surgical discussion

Veterinary studies concerning the surgical management of CVM are reserved to atlantoaxial instability [36–40]. A direct comparison of surgical techniques cannot be made due to different follow-up periods and outcome measures [36]. Other uncontrolled factors influence outcome such as the surgeon’s experience, initial neurological status, age of the dog and chronicity of neurological signs [36].

In the human literature controversy persists regarding the best surgical approach for management of cervical malformations. Anterior decompression by a transoral approach or mandibular split is indicated if spinal cord compression occurs from an anterior position. This approach is optimal for decompression of the cervicomedullary junction [27]. However, compared to the posterior approach, the anterior approach has disadvantages, including a more complicated technique, higher invasiveness, difficulty in primary fixation, higher incidence of postoperative infection, and increased incidence of respiratory tract disorders [27]. Jones and colleagues [41] reported that 7 of 44 patients that underwent an anterior approach, had complications such as dysphagia, nasal regurgitation and infection. In contrast, posterior approaches are increasingly more popular because osseous compression can be resolved by the reduction of abnormally positioned bone without the above-mentioned complications. However, spinal cord decompression could be insufficient if the reduction is not successful [41].

Grosso and colleagues [42] reported that 25% of the human patients suffered with at least one perioperative complication. The most common perioperative complications were thrombosis (10.5%), deep wound infection (7.9%), pneumonia (5.2%), pulmonary embolism (3.9%), postoperative hematoma (3.9%) and dysphagia (2.6%). The overall mortality rate was 4%. Perioperative complication rates with combined ventral and dorsal procedures were higher (40%) compared to ventral-alone (30%), and dorsal-alone (27%) procedures. Implant malposition was present in 5.3% of patients. Sixteen percent of patients underwent revision surgery, which includes 5.2% of patients who underwent revision because of infection. Implant malposition occurred in 10% of patients who underwent the dorsal approach, 10% who underwent the ventral, and 2.5% of patients who underwent the combined approach. Revision surgery was needed in 15, 10, and 18% of cases for the dorsal, ventral, and combined approaches, respectively. In our cases series, no immediately post-operative complications were reported. In case 7, implant failure led to revision surgery which was considered successful. The relapse of the clinical signs 323 days after the initial presentation was attributed to the chondrodystrophic nature of the breed and predisposition to intervertebral disc disease, rather than adjacent segment disease. Grosso found that the ventral approach had similar degrees of reduction, lower complication rates, and possibly greater neurological improvement when compared to the combined approach [42]. These results suggest that a ventral approach alone is an effective method for surgical correction of cervical malformations, and may be safer than the combined approach. This fact could be observed in our population diagnosed with atlantoaxial pseudoarthrosis, where case 2 required a ventral stabilisation after a dorsal decompression (Fig. 1d) while in case 1 a ventral stabilisation alone resulted in resolution of the clinical signs (Fig. 1e). Given the heterogeneity and the limited number of cases in this study, it is difficult to compare ventral and combined approaches. Further studies would be required to determine statistical relevance regarding the relative success of surgical approaches.

The major limitations of this study are its retrospective nature and the limited number of cases. The lack of standardisation of radiological investigations and follow-ups are also limitations as different follow-ups performed at different timings may have resulted in different outcomes. Dynamic studies were not performed in all cases. Some patients were followed-up with radiographs only while others underwent MR or CT scanning which, again may have contribute to a different outcome. The heterogeneity of the population studied also have contributed for the different clinical approaches of the veterinary surgeons responsible for each case.

## Conclusion

Cervical vertebral malformations are rare anomalies that still poorly characterised in veterinary medicine. Knowledge on how to recognize and treat cervical anomalies in the canine population is important to prevent possible neurologic complications. In addition, anatomic anomalies of the cervical spine in veterinary patients often coexist with skeletal dysplasia and connective tissue disorders and other genetically inherited metabolic disorders. Klippel-Feil like syndrome in dogs appeared to have a good long-term outcome with conservative management in medium to large breed dogs. Vertebral instability and spinal cord compression were indications for surgical management. Indications for a ventral versus a combined ventral and dorsal approach remain in need of better definition, although a ventral approach appeared to be safe. Imaging of the entire vertebral column, including dynamic studies is recommended when CVM is diagnosed.

## Abbreviations

3D: Three dimensional
CARD: Congenital atlantoaxial rotatory displacement
CCF: Congenital cervical fusion
CT: Computed tomography
CVM: Cervical vertebral malformations
DCM: Degenerative cervical myelopathy
DISH: Diffuse idiopathic skeletal hyperostosis
ECVN: European college of veterinary neurology
ECVS: European college of veterinary surgeons
ECVSMR: European college of veterinary sports medicine and rehabilitation
KFS: Klippel Feil-like syndrome
MR: Magnetic resonance
PMMA: Polymethylmethacrylate
STIR: Short-tau inversion recovery
T2W: T2-weighted

## References

[1] Giampietro PF, Raggio CL, Blank RD, McCarty C, Broeckel U, Pickart MA (2013). Clinical, genetic and environmental factors associated with congenital vertebral malformations. Mol Syndromol.

[2] Klimo P, Rao G, Brockmeyer D (2007). Congenital anomalies of the cervical spine. Neurosurg Clin N Am.

[3] Kim HJ (2013). Cervical spine anomalies in children and adolescents. Curr Opin Pediatr.

[4] Menezes AH (2008). Craniocervical developmental anatomy and its implications. Childs Nerv Syst.

[5] Viallet P (1951). Two cases of cervical localization of Baastrup disease. J Radiol Electrol.

[6] Goto S, Tanno T, Moriya H (1995). Cervical myelopathy caused by pseudoarthrosis between the atlas and axis associated with diffuse idiopathic skeletal hyperostosis. Spine..

[7] Kohler A, Zimmer EA, Wilk SP (1968). Upper cervical spine. Borderlands of the normal and early pathologic in skeletal roentgenology.

[8] Suzuki T, Miyamoto H, Sumi M, Inui Y, Uno K, Takabatake M, Tadokoro K (2008). Cervical myelopathy caused by pseudoarthrosis between posterior arch of the atlas and lamina of the axis in Klippel-Feil syndrome: a case report. J Spinal Disord Tech.

[9] Miyakoshi N, Hongo M, Kasukawa Y, Shimada Y (2010). Cervical myelopathy caused by atlas Osteochondroma and Pseudoarthrosis between the Osteochondroma and Lamina of the Axis. Case report. Neurol Med Chir.

[10] Pang D, Li V (2004). Atlantoaxial rotatory fixation: part 1 – biomechanics of normal rotation at the atlantoaxial joint in children. Neurosurgery.

[11] Reber K, Bürki A, Reves NV, Stoffel M, Gendron K, Ferguson SJ, Forterre F (2013). Biomechanical evaluation of the stabilizing function of the atlantoaxial ligaments under shear loading: a canine cadaveric study. Vet Surg.

[12] Spiegel D, Shrestha S, Sitoula P, Rendon N, Dormans J (2017). Atlantoaxial rotatory displacement in children. World J Orthop.

[13] Samartzis D, Shen FH, Herman J, Mardjetko SM (2010). Atlantoaxial rotatory fixation in the setting of associated congenital malformations: a modified classification system. Spine.

[14] Pang D, Li V (2005). Atlantoaxial rotatory fixation: part 2 – new diagnostic paradigm and a new classification based on motion analysis using computed tomographic imaging. Neurosurgery.

[15] Pang D, Li V (2005). Atlantoaxial rotatory fixation: part 3 – a prospective study of the clinical manifestation, diagnosis, management and outcome of children with atlantoaxial rotatory fixation. Neurosurgery.

[16] Samartzis D, Herman J, Lubicky JO, Shen FH (2006). Classification of congenitally fused cervical patterns in Klippel-Feil patients: epidemiology and role in the development of cervical spine-related symptoms. Spine.

[17] Xue X, Shen J, Zhang J, Tian Y, Hong Zhao H, Yipeng Wang Y, Jinqian Liang J, Li Z, Qiu G (2014). Klippel-Feil Syndrome in congenital scoliosis. Spine.

[18] Gruber J, Saleh A, Bakhsh W, Rubery PT, Addisu Mesfin A (2018). The prevalence of Klippel-Feil syndrome: a computed tomography based analysis of 2,917 patients. Spine Deformity.

[19] Bertolini G, Trotta M, Caldin M (2015). A skeletal disorder in a dog resembling the Klippel–Feil syndrome with Sprengel’s deformity in humans. J Small Anim Pract.

[20] Samartzis D, Kalluri P, Herman J, Lubicky JP, Shen FH (2008). The extent of fusion within the congenital Klippel-Feil segment. Spine.

[21] Samartzis D, Jean Herman J, Lubicky JO, Shen FH (2007). Sprengel’s Deformity in Klippel-Feil Syndrome. Spine.

[22] Sabuncuoglu H, Ozdogan S, Karadag D, Timurkaynak E (2011). Congenital hypoplasia of the posterior arch of the atlas: case report and extensive review of the literature. Turkish Neurosurgery.

[23] Dokai T, Nagashima H, Nanjo Y, Tanida A, Teshima R (2011). Posterior occipitocervical fixation under skull-femoral traction for the treatment of basilar impression in a child with Klippel–Feil syndrome. J Bone Joint Surg Br.

[24] Ogihara N, Takahashi J, Hirabayashi H, Mukaiyama K, Kato H (2013). Surgical treatment of Klippel–Feil syndrome with basilar invagination. Eur Spine J.

[25] Chau AMT, Wong JH, Mobbs RJ (2009). Cervical myelopathy associated with congenital C2/3 canal stenosis and deficiencies of the posterior arch of the atlas and laminae of the Axis: case report and review of the literature. Spine.

[26] Sharifi G, Lotfinia M, Rahmanzade R, Lotfinia AA, Rahmanzadeh R, Omidbeigi M (2018). Congenital absence of the posterior element of C1, C2 and C3 along with bilateral absence of C4 pedicles: case report and review of the literature. World Neurosurgery.

[27] Sahoo S, Salunke P (2017). C2 body as the ‘keystone’ in management of C1-C2-C3 dislocation secondary to congenital absence of posterior element: a case report. World Neurosurgery.

[28] Finn MA, MacDonald JD. C2–3 anterior cervical fusion: technical report. BSD Journal of Spinal Disorders and Techniques Publish Ahead of Print. 10.1097/BSD.0b013e318292b3ca.

[29] De Rycke L, Saunders JH (2017). Congenital anomalies of the vertebrae in dogs. Vlaams Diergeneeskundig Tijdschrift..

[30] Chandraraj S (1987). Failure of articular process (zygapophyseal) joint development as a cause of vertebral fusion (blocked vertebrae). J Anat.

[31] Bailey CS, Morgan JP (1992). Congenital spinal malformations. Vet Clin North America: Small Anim Pract.

[32] Nouri A, Martin AR, Lange SF, Kotter M, Mikulis DJ, Fehlings MG (2017). Congenital cervical fusion as a risk factor for development of degenerative cervical myelopathy. World Neurosurgery.

[33] Song KJ, Choi BW, Jeon TS, Lee KB, Chang H (2011). Adjacent segment degenerative disease: is it due to disease progression or a fusion-associated phenomenon? Comparison between segments adjacent to the fused and non-fused segments. Eur Spine J.

[34] Ortega M, Gonçalves R, Haley A, Wessmann A, Penderis J (2012). Spondylosis deformans and diffuse idiopathic skeletal hyperostosis (dish) resulting in adjacent segment disease. Vet Radiol Ultrasound.

[35] Ryan R, Gutierrez-Quintana R, Ter Haar G, De Decker S (2017). Prevalence of thoracic vertebral malformations in French bulldogs, Pugs and English bulldogs with and without associated neurological deficits. Vet J.

[36] Stalin C, Gutierrez-Quintana R, Faller K, Guevar J, Yeamans C, Penderis J (2015). A review of canine atlantoaxial joint subluxation. Vet Comp Orthop Traumatol.

[37] Thomas WB, Sorjonen DC, Simpson ST (1991). Surgical management of atlantoaxial subluxation in 23 dogs. Vet Surg.

[38] Beaver DP, Ellison GW, Lewis DD (2000). Risk factors affecting the outcome of surgery for atlantoaxial subluxation in dogs: 46 cases (1978-1998). J Am Vet Med Assoc.

[39] Aikawa T, Shibata M, Fujita H (2013). Modified ventral stabilization using positively threaded profile pins and polymethylmethacrylate for atlantoaxial instability in 49 dogs. Vet Surg.

[40] Galban EM, Gilley RS, Long SN (2010). Surgical stabilization of an occipitoatlantoaxial malformation in an adult dog. Vet Surg.

[41] Jones DC, Hayter P, Vaughan D, Findley GFG (1998). Oropharyngeal morbidity following transoral approaches to the upper cervical spine. Int J Oral Maxillofac Surg.

[42] Grosso MJ, Hwang R, Krishnaney AA, Mroz TE, Benzel EC, Steinmetz MP (2015). Complications and outcomes for surgical approaches to cervical kyphosis. J Spinal Disord Tech.

